# The Effect of Perioral Scan and Artificial Skin Markers on the Accuracy of Virtual Dentofacial Integration: Stereophotogrammetry Versus Smartphone Three-Dimensional Face-Scanning

**DOI:** 10.3390/ijerph18010229

**Published:** 2020-12-30

**Authors:** Hang-Nga Mai, Du-Hyeong Lee

**Affiliations:** 1 Institute for Translational Research in Dentistry, Kyungpook National University, Daegu 41940, Korea; maihangnga1403@knu.ac.kr; 2 Department of Prosthodontics, School of Dentistry, Kyungpook National University, Daegu 41940, Korea

**Keywords:** intraoral scanners, face scanners, perioral scans, skin markers, accuracy, dentofacial integration, stereophotogrammetry, smartphone, three-dimensional face-scanning

## Abstract

This study evaluated the effects of different matching methods on the accuracy of dentofacial integration in stereophotogrammetry and smartphone face-scanning systems. The integration was done (N = 30) with different matching areas (*n* = 10), including teeth image only (TO), perioral area without markers (PN) and with markers (PM). The positional accuracy of the integrated models was assessed by measuring the midline linear deviations and incisal line canting between the experimental groups and laser scanner-based reference standards. Kruskal–Wallis and Mann–Whitney U tests were used for statistical analyses (α = 0.05). The PM method exhibited the smallest linear deviations in both systems; while the highest deviations were found in the TO in stereophotogrammetry; and in PN in smartphone. For the incisal line canting; the canting degree was the lowest in the PM method; followed by that in the TO and the PN in both systems. Although stereophotogrammetry generally exhibited higher accuracy than the smartphone; the two systems demonstrated no significant difference when the perioral areas were used for matching. The use of perioral scans with markers enables accurate dentofacial image integration; however; cautions should be given on the accuracy of the perioral image obtained without the use of markers.

## 1. Introduction

The assessment of facial and dental structures is essential for successful esthetic prosthodontic treatments [1,2]. In order to achieve predictable esthetic outcomes, the two-dimensional (2D) esthetic analysis that uses facial and dental photographs is introduced [3]. In the 2D analysis, full-face photography at rest, wide smile, and lip-retracted states is imported to the image editing software where the patient’s facial and dental characteristics are analyzed with respect to the facial reference lines [4]. Preferred teeth shapes can be recommended using the sketching function on the digital photographs. The digital 2D smile design provides a visual guide for esthetic rehabilitation planning from a facial perspective; however, difficulty in transferring the proposed design to the actual operation field remains a major concern [5,6,7].

With the development of three-dimensional (3D) facial scanning, it has become possible to virtually plan a prosthetic design in harmony with the corresponding facial appearance in a 3D view [8,9]. The 3D facial scan enables the creation of a virtual face that can be integrated with 3D dental images obtained from digital scanning of the dentition [8,10]. For the 3D dentofacial integration, the 3D facial scans are obtained with anterior teeth exposed [11]. Subsequently, the teeth area visible on the facial scan images are used as a reference to match the facial scans with intraoral scans [10,12]. The accuracy of the image integration mainly depends on the spatial accuracy and the resolution of the captured anterior teeth image in the digital face scans [12].

High-end stationary stereophotogrammetry and laser-based face-scanning systems are commonly used in different fields related to facial rehabilitation, orthopedic, and plastic surgery [13]. However, most professional face-scanning systems are expensive or complex and often require a lengthy training period for learning the complicated scanning protocols [14,15,16]. In recent decades, the use of smartphones with 3D depth sensor cameras that use structured light or time of flight technology for facial digitization has been attracting increasing interest owing to the high portability, user friendliness, cost-effectiveness, and popularity of mobile devices [11,17,18,19]. The advantages of smartphone face digitization include reduce time for scanning, image processing, and technical learning [20,21]. Several smartphone applications have been developed for scanning faces and rendering 3D facial models that can be transferred to a dental computer-aided design (CAD) software [11,18]. The integration of smartphone 3D face scan data with prosthesis designing is reportedly effective for communication between patients and dental specialists because of the direct visualization of the expected outcomes and the immediate design modification of the prosthesis as per patient preference in the CAD stage [11,17,19].

The incorporation of 3D face scan to digital prosthetic workflow facilitates esthetic and predictable treatments. Whereas, the dentofacial image integration process remains challenging owing to the limited area for image matching and the quality of tooth image in the facial scan. In order to improve the accuracy of dentofacial integration, additional perioral scanning while performing intraoral scanning is introduced, particularly in edentulous patients who have missing maxillary anterior teeth [22]. Although a perioral scan provides a larger matching area for the image fusion, the effects of this method on the accuracy of the image matching remain to be verified. Therefore, this study aimed to evaluate the effects of the use of different reference images, including teeth image only, perioral image obtained without artificial markers, and perioral image obtained with artificial markers on the accuracy of virtual dentofacial integration in stereophotogrammetry and smartphone face-scanning systems. The first null hypothesis was that the use of perioral scan and artificial skin markers would not influence the accuracy of 3D dentofacial integration. The second null hypothesis was that the accuracy of 3D dentofacial integration would be comparable in the stereophotogrammetry and smartphone face-scanning systems.

## 2. Materials and Methods

### 2.1. Study Design

We enrolled an adult volunteer with full anterior teeth, no craniofacial syndrome or deformities, no face scar tissue, and no history of facial trauma and maxillofacial surgery in this study. This volunteer was explained the study procedure and signed a written informed consent before data collection.

### 2.2. Facial Image Acquisition

**Figure 1:**
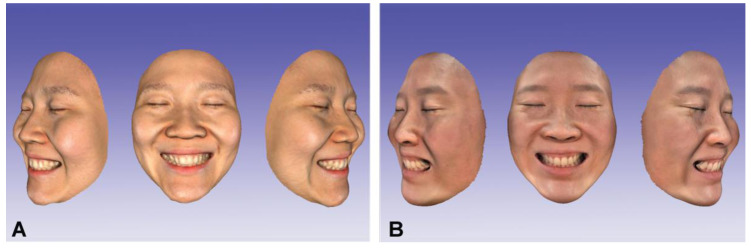
Three-dimensional face scans of the face with anterior teeth in broad smile (e phoneme) with different face-scanning systems. (**A**) Stereophotogrammetry; (**B**) smartphone depth sensor camera.

For image acquisition of the full face with anterior teeth in the broad smile position, the participant was instructed to smile while pronouncing the [ē] sound (e phoneme) [23]. The facial scans were taken with a stationary stereophotogrammetric system that comprised four digital cameras (Canon EOS 100D with Canon EF LENS 50 mm f1.8 STM, Canon Corp., Tokyo, Japan) with a 3D image reconstruction software (Di3D capture v.6,8.17.4490, Dimensional Imaging, Glasgow, Scotland, UK), and a smartphone 3D depth sensor camera (iPhone X; Apple Store, Cupertino, CA, USA) with a face-scanning application for dental use (Bellus3D, version 1.8.6, Bellus3D, Inc. Campbell, CA, USA) (Figure 1). Before face scanning, the participant was instructed to remove all makeup, accessories, glasses, and to use hair bands to tighten up hair. For stereophotogrammetric face scanning, the participant was required to maintain an immobile sitting position, with the head upright and the occlusal plane of the maxillary teeth parallel to the floor. The face was illuminated by commercial white-light studio flash units (Speedlite 430 EX II; Canon UK Ltd., Woodhatch, Reigate Surrey, UK) that were connected to the cameras. The cameras were set for ISO sensitivity 100, shutter speed 1/200 s, and focal ratio (aperture) 20 with a focal length of 50 mm, and the resolution of the cameras is 3888 pixels × 2592 pixels [24]. For the smartphone face scanning, the smartphone was fixed at a distance of 30 cm using a smartphone holder stand. The participant was scanned by rotating the head to the right and the left of the camera following the manufacturer’s instructions while maintaining the head at the center of the camera. The scanning procedures were performed under the indoor environment with room light conditions in 15 s, and the scanning mode was set in high-definition (HD mode) in the scanning software [25]. The noise image data on the acquired 3D face models including the hair, ears, and nostrils were trimmed and saved in the wavefront object (OBJ) file format.

### 2.3. Dental Image Acquisition

**Figure 2:**
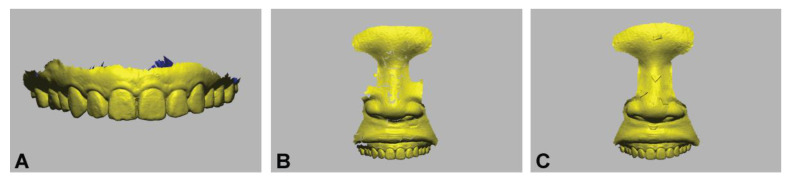
Dental image acquisition conducted with an intraoral optical scanner. (**A**) Intraoral scan; (**B**) intraoral and perioral scans obtained without an artificial marker; (**C**) intraoral and perioral scans obtained with artificial markers.

The intraoral and perioral anatomical structures were digitized using an intraoral optical scanner (CS3600, Carestream, Rochester, NY, USA) (Figure 2) [22]. For the full-arch scanning of the maxilla, the zig-zag scanning technique was used [26]. Starting from the right quadrant, the tip of the scanner drew an arc movement along the dental arch, from vestibular to the palatal area, slowly moving forward. The perioral structures, including the upper lip, philtrum, and nose were obtained with the participant’s anterior teeth in a broad smile (e phoneme) position. The perioral scans were performed twice with and without the use of artificial skin markers. A single proficient clinician performed all the scan processes, and the scanned images were stored as separated standard triangle language (STL) files.

### 2.4. Dentofacial Image Integration

**Figure 3:**
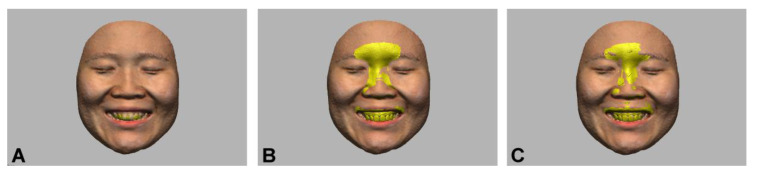
Dentofacial image integration performed using different image matching methods. (**A**) teeth image only; (**B**) teeth and perioral area without marker; (**C**) teeth and perioral area with markers.

The 3D dentofacial image integration was performed by registering the dental scans to the facial scans in the image control computer software (Geomagic Design X; 3D Systems, Rock Hill, SC, USA). The point-based best-fit algorithm was used for the global registration. According to the image matching areas and the dental scans used for the image registration, the following three experimental groups were defined: teeth image only (TO), perioral area without marker (PN), and perioral area with marker (PM). In the TO group, three anatomic landmarks, including the proximal contact area between teeth 11 and 21, the cusp tip of teeth 13 and 23, were selected as the fiducial points [27]. In the PN and PM groups, the nasal structure in the perioral image was used for the image matching in addition to the dental landmarks presented in the TO group (Figure 3) [22]. The image integrations were conducted ten times in each group by a single experienced operator who was blinded to the purpose of this study.

#### Construction of the Reference Image and the 3D Coordinate System

**Figure 4:**
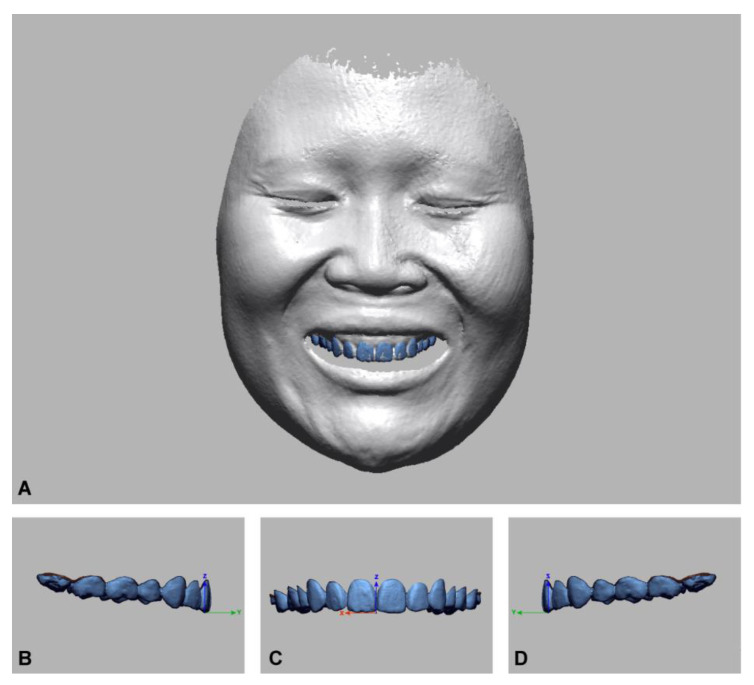
Construction of the reference image and the establishment of spatial coordinate system. (**A**) reference image; (**B**) left view of the coordinate system; (**C**) front view of the coordinate system; (**D**) right view of the coordinate system.

**Figure 5:**
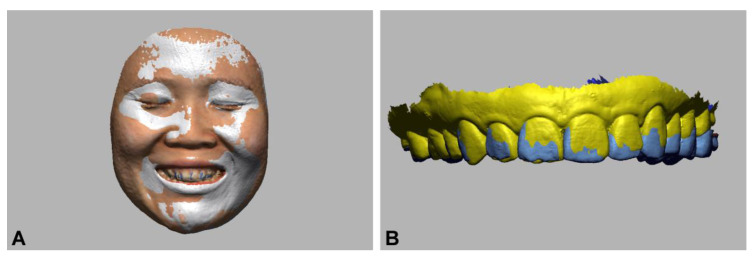
(**A**) Orientation of the integrated dentofacial model into the reference image; (**B**) dental images for accuracy evaluation.

In this study, a reference image was constructed by combining the intraoral scan with a high-resolution 3D facial model obtained with an industrial laser scanner (MetraSCAN-R; Creaform, Levis, QC, Canada) with the corresponding software (VXelements v.7.0.3, Creaform, Lévis, QC, Canada) (Figure 4A) [12]. The 3D coordinate system of reference image was established using the image control computer software (Geomagic Design X, 3D Systems Inc., Rock Hill, SC, USA). The X-axis of the coordinate system was set for the horizontal measurement in the frontal plane; the Y-axis was for the horizontal measurement in the sagittal plane, and the Z-axis was for the vertical measurement in the frontal plane (Figure 4B–D). The dentofacial images from the experimental groups were oriented to the reference image for unification into the same coordinate system using the immobile face structures as the references (Figure 5A) [28]. After the registration, the face image was deleted, leaving the dental images for the evaluation of the accuracy of dentofacial integration (Figure 5B).

### 2.5. Evaluation of the Spatial Position of the Integrated Dental Images

**Figure 6:**
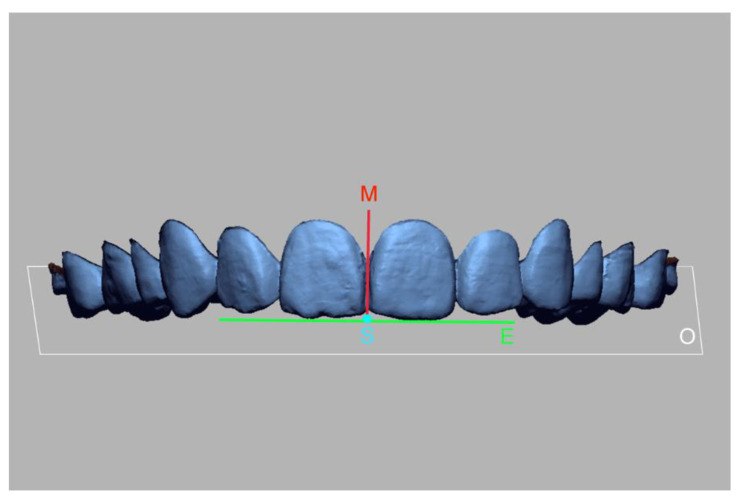
Landmarks for the evaluation of the spatial positional accuracy of the dentofacial integration. M, interincisal midline; O, occlusal plan; S, intersection point of the midline and the occlusal plan; E, incisor edge line.

The spatial positional accuracy of the dentofacial integrated images was assessed by measuring the displacements of the maxillary on X, Y, and Z axes (Figure 6). The maxillary interincisal midline (line M) and occlusal plane (plan O) were selected, and the point of intersection (point S) was determined. The midline linear deviations in the horizontal, antero-posterior, and vertical dimensions were defined as the distances between the point S in the integrated dental models and the reference image on the X-axis, Y-axis, and Z-axis, respectively. Angular deviations of the incisal line were determined by calculating the shift angle between the central incisor edge lines (line E) of the maxillary between the reference and experimental groups. The midline linear deviations were obtained using the measurement of linear distance between the points command, and the incisal line canting was calculated by applying the measurement of angle between the lines command of the software (Geomagic Design X). The measurement results were recorded for analyzing the precision of the integrated dento-facial images.

### 2.6. Statistical Analyses

The Shapiro–Wilk test for verifying the normality of distribution showed that the PN and PM groups in stereophotogrammetry, and the PM group in smartphone face scanning had *p* < 0.05, which means the datasets were not in normal distribution. In addition, the Levene test for evaluating the equality of data variances revealed that the assumption of equal variances was met for the data in stereophotogrammetry (*p* = 0.068) and smartphone face scanning (*p* = 0.145). Thus, nonparametric tests, Kruskal–Wallis test and Mann–Whitney U tests, were used to analyze the differences among the groups in terms of linear and incisal line canting deviations. All the statistical analyses were performed with SPSS v25.0 statistical software (IBM Corp., Armonk, NY, USA). Statistical significance was set at a *p*-value < 0.05.

## 3. Results

**Figure 7:**
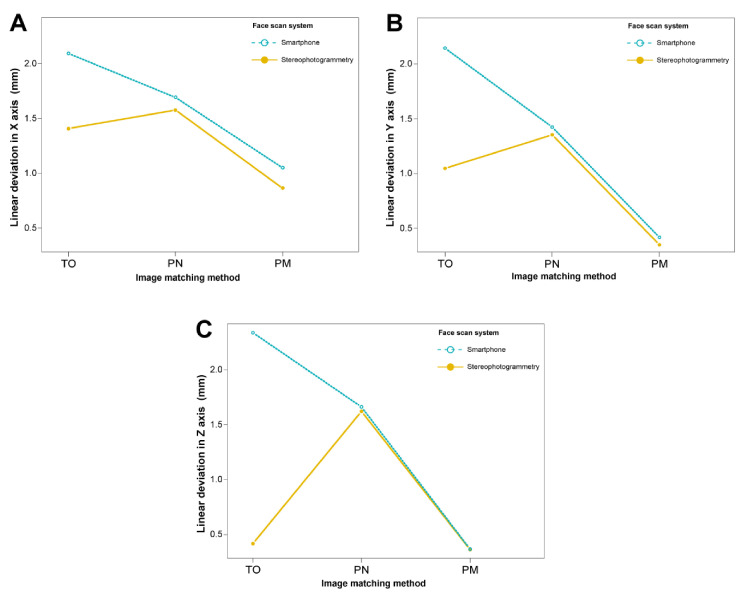
Linear deviations in the image registration of intraoral scan to facial scan. (**A**) in X-axis; (**B**) in Y-axis; (**C**) in Z-axis. TO, teeth image only; PN, perioral image without marker; PM, perioral image with markers.

Outcomes of the linear deviation in the X, Y, and Z axes are represented in Table 1 and Figure 7. The mean linear deviation was smallest in the PM method, followed by those in the PN and TO methods in the stereophotogrammetry. In the smartphone face scanning, the mean linear deviation was smallest in the PM method, followed by those in the TO and PN methods. No significant difference was found between the stereophotogrammetry and smartphone face scanning in the PM and PN methods; meanwhile, in the TO method, the linear deviation was smaller in the stereophotogrammetry than in the smartphone face scanning, especially in the Y and Z axes (*p* = 0.008).

**Figure 8:**
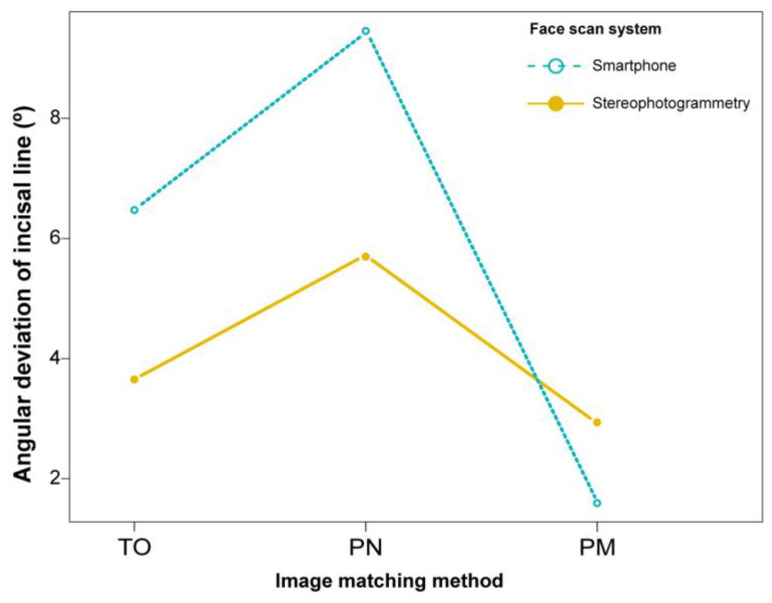
Angular deviations of the incisal line in the image registration of intraoral scan to facial scan. TO, Teeth image only; PN, perioral image without marker; PM, perioral image with markers.

Outcomes of the angular deviation of the incisal line are shown in Table 2 and Figure 8. The mean angular deviation was least in the PM method, followed by those in the TO and PN methods in both stereophotogrammetry (*p* = 0.004) and smartphone face scanning (*p* = 0.005). In a comparison between the devices, stereophotogrammetry showed smaller angular deviations than smartphone face scanning in the TO and PN methods (*p* = 0.008 and *p* = 0.151, respectively). However, in the PM method, the mean angular deviation was smaller in smartphone face scanning than in stereophotogrammetry (*p* = 0.008).

## 4. Discussion

Based on the results of this study, the use of perioral scan with artificial skin markers significantly improved the accuracy of integration of dental model to facial scan. When the perioral scan was used for image matching without a marker, the matching deviations were larger than the results obtained using markers, and even larger than those obtained using only teeth images in the stereophotogrammetry. Thus, the first null hypothesis that the use of perioral scan and artificial skin markers would not affect the accuracy of 3D dentofacial integration, was rejected. Interestingly, the stereophotogrammetry face-scanning system exhibited higher accuracy than the smartphone face-scanning system when only teeth images were used for matching; however, there was no difference when the perioral areas were used for image matching. Therefore, the second null hypothesis that the accuracy of 3D dentofacial integration would not be different in the stereophotogrammetry and smartphone face-scanning systems, was partially rejected.

With the increase in the use of 3D facial scanning in prosthodontic treatment planning, it is necessary to validate the accuracy of the integrated dentofacial images. When a face is digitally scanned, the 3D image of the face is reconstructed on a digital wireframe that consists of polygons [29,30]. The accuracy of digitization could be influenced by the accuracy of point clouds created and the resultant polygons that are computed by the software algorithm based on the visibility of the scanned structures [12]. Precise and small polygons can be constructed from the well visualized parts of the face, resulting in higher resolution 3D images. In contrast, less visible structures, such as the teeth positioned at the corners of the mouth or teeth overlapped by the lips, are reconstructed with inaccurate and larger polygons that lead to image deformation, distortion, and warping. Moreover, the shinning surfaces of the teeth and gingiva may lead to inaccurate image capture due to the light reflection. Thus, when only the teeth area was used for image matching between the face scan and intraoral scan images, the matching could be prone to error because of the image deformations of the 3D facial model at the mouth region owing to the difficulties in capturing the complex structures of the teeth and the gingiva [12]. In the smartphone 3D depth camera face scan, the teeth scan quality could be inferior to that of the stereophotogrammetry because of the high sensitivity to the depth of the smartphone face scan [31]. The smartphone-base facial models are reconstructed by compiling the 3D facial images to create a depth map of the object and the surroundings [32]. The system uses a dot projector to project infrared dots on the object during a scan. The dot pattern appears geometrically distorted following the surface shape of the scanned objects. A proximity sensor in the camera of the system receives the reflected dot light and obtains surface depth of the object by analyzing the deformation of the dot pattern. Subsequently, a 3D model of the scanned object is built with a dedicated face-scanning smartphone application based on the depth information. By the working principle, regions with low depth contrast on the objects, such as central incisors may be difficult to reconstruct with high depth resolution; in contrast, regions with overly difference in depth and/or in orientation on the faces, such as the canines, may be distorted [33].

In order to improve the accuracy of the dentofacial integration, the intraoral scan of the teeth area together with the perioral structures has been suggested [22]. The objective of using this approach is to provide larger areas that can be used as a reference to align the intraoral scan of the teeth with the 3D scan of the face. Interestingly, the results of our study showed that the effect of perioral scan method on image matching depends on the use of artificial markers during the perioral scanning. The possible reason is attributed to the inaccuracy of the scan data obtained by the intraoral scanner when capturing the large areas of the perioral structures without the skin marker attachment. When performing 3D scans, an intraoral scanner utilizes various morphological characteristics from the target object as a reference when stitching the scan images together [34,35]. This process is accurate if the surface of the target objet is morphologically distinct. However, for large featureless surface, difficulties arise in the stitching process because of the lack of reference image landmarks. The scanning accuracy of the intraoral scanners is considerably influenced by the distinguishability of the objects and the size of the scanning area [36,37,38]. It could be problematic for the intraoral scanners to correctly digitize the lip, philtrum, and nose because the structures do not have small and characteristic anatomic features in shape. The lack of clear landmarks on soft tissues makes it difficult to perform image stitching between individual capture images; thereby, incurring inaccurate scan results such as image distortion or discontinuity [39]. Artificial markers supply distinct references from featureless adjacent areas, so could help the image stitching process. In this study, the use of markers positioned on the non-specific shape of soft tissues was believed to enhance the accuracy of the perioral scan method. The artificial markers not only supplied additional landmarks for correct image stitching but also facilitated the image acquisition process for obtaining a digital impression. The number and position of artificial markers can be related with the size of scanner head. A larger scanner head obtains a bigger size of single shots, which increases the chances of capturing the distinct area in the shots [40]. Thus, less number of artificial makers might be needed, and the markers can be placed more widely. The shape of markers could also influence the accuracy of image stitching. Effective shape and size of markers should be investigated to reduce the faulty processing of digitization in further studies. In addition, the scan strategy, image reconstruction software, operator experience are another major factors influencing the quality of a scan [41,42,43].

Differences in the virtual integrated dentofacial model and the actual face lead to an inaccurate esthetic prognosis because the 3D image models are used for planning the medical and dental treatments. Maxillary midline deviations and incisal line canting are essential factors for assessing the smile esthetics [44]. In a clinical context, discrepancies of <2 mm for midline deviations and <3° for frontal incisal line canting appear to have a less noticeable impact on dentofacial esthetics [45]. In the present study, the midline deviations were within 2 mm for all the matching methods in both the two face-scanning systems; this was clinically acceptable. The frontal incisal plane canting was within 3° when the teeth and perioral with the markers were used; however, when the perioral without marker was used, the canting degree was increased up to 9°. Based on the results, special caution should be given when the perioral image that was taken without artificial markers was to be used as a fiducial matching region for dentofacial integration. The use of skin markers is recommended for enhancing the accuracy of image capturing in the perioral areas.

The first limitation of this study was the relatively small sample size. Although this pilot study presented the linear tendency in results, further clinical studies with larger sample sizes are required to extend the application and confirm the impact of the present study. Second limitation relates to the lack of evaluation of the posterior part of the dentition. As the dentofacial image is mainly used for assessing the smile and esthetic treatments, we focused on the anterior region of the oral cavity. Lastly, considering the prolonged scanning time in the mobile scanning method, errors could arise by involuntary facial movements. Thus, patient-related factors should also be included in further studies.

## 5. Conclusions

The accuracy of virtual dentofacial integration was largely depended on the use of perioral scans and artificial skin markers. The smallest midline deviation and frontal plan canting were found when the perioral image with artificial markers was used, while the highest deviations were found when the perioral image obtained without marker was used for image matching. Although stereophotogrammetry face scan generally exhibited higher accuracy of virtual dentofacial integration than the smartphone 3D depth camera face scan, the difference between the devices was not significant when the perioral scans were used as references for image matching.

## Figures and Tables

**Table 1:** Mean (standard deviation) of linear deviations in the image registration of intraoral scan to facial scan in the X, Y, and Z axes.

Axis	Face Scan System	TO	PN	PM	*p*-Value
X	Stereophotogrammetry	1.409 (0.291) ^a,1^	1.577 (0.365) ^a,1^	0.865 (0.215) ^b,1^	0.027
	Smartphone	2.091 (0.750) ^a,1^	1.691 (0.084) ^a,1^	1.051 (0.341) ^b,1^	0.027
	*p*-value	0.151	0.69	0.421	
Y	Stereophotogrammetry	1.045 (0.074) ^a,1^	1.351 (0.027) ^b,1^	0.351 (0.047) ^c,1^	0.002
	Smartphone	2.139 (0.162) ^a,2^	1.420 (0.348) ^b,1^	0.418 (0.094) ^c,1^	0.002
	*p*-value	0.008	0.151	0.31	
Z	Stereophotogrammetry	0.417 (0.406) ^a,1^	1.623 (0.026) ^b,1^	0.363 (0.071) ^a,1^	0.006
	Smartphone	2.340 (0.333) ^a,2^	1.663 (0.143) ^b,1^	0.367 (0.040) ^c,1^	0.002
	*p*-value	0.008	0.151	0.548	

^a,b,c^ Superscript alphabetical letters in the same row indicate a significant difference between matching methods; ^1,2^ Superscript numbers in the same column indicate a significant difference between face-scanning systems; TO: teeth image only; PN: perioral image without marker; PM: perioral image with markers. Kruskal–Wallis and Mann–Whitney U tests were used for statistical analyses (α = 0.05).

**Table 2:** Mean (standard deviation) of angular deviations of the incisal line (°) in the image registration of intraoral scan to facial scan.

Face Scan System	TO	PN	PM	*p*-Value
Stereophotogrammetry	3.652 (0.531) ^a,1^	5.722 (1.678) ^b,1^	2.926 (0.159) ^a,1^	0.004
Smartphone	6.490 (0.739) ^a,2^	9.441 (3.339) ^b,1^	1.578 (0.718) ^a,2^	0.005
*p*-value	0.008	0.151	0.008	

^a,b,c^ Superscript alphabetical letters in the same row indicate a significant difference between matching methods; ^1,2^ superscript numbers in the same column indicate a significant difference between face-scanning systems; TO: teeth image only; PN: perioral image without marker; PM: perioral image with markers. Kruskal–Wallis and Mann–Whitney U tests were used for statistical analyses (α = 0.05).

## Data Availability

Data is contained within the article.
